# Lack of quadruple and quintuple mutant alleles associated with sulfadoxine-pyrimethamine resistance in *Plasmodium vivax* isolates from Brazilian endemic areas

**DOI:** 10.1590/0074-02760180425

**Published:** 2019-02-04

**Authors:** Larissa Rodrigues Gomes, Aline Lavigne, Patrícia Brasil, Cassio Leonel Peterka, Didier Ménard, Cláudio Tadeu Daniel-Ribeiro, Maria de Fátima Ferreira-da-Cruz

**Affiliations:** 1 Fundação Oswaldo Cruz-Fiocruz, Instituto Oswaldo Cruz, Laboratório de Pesquisa em Malária, Rio de Janeiro, RJ, Brasil; 2 Fundação Oswaldo Cruz-Fiocruz, Centro de Pesquisa, Diagnóstico e Treinamento em Malária, Rio de Janeiro, RJ, Brasil; 3 Ministério da Saúde, Secretaria de Vigilância em Saúde, Programa Nacional de Prevenção e Controle da Malária, Brasília, DF, Brasil; 4 Fundação Oswaldo Cruz-Fiocruz, Instituto Nacional de Infectologia Evandro Chagas, Laboratório de Doenças Febris Agudas, Rio de Janeiro, RJ, Brasil; 5 Institut Pasteur, Malaria Genetic and Resistance Group, Biology of Host-Parasite Interactions Unit, Paris, France

**Keywords:** P. vivax, malaria, pvdhfr, pvdhps, chemoresistance

## Abstract

**BACKGROUND AND OBJECTIVE:**

Brazil is responsible for a large number of *Plasmodium vivax* cases in America. Given the emergence of *P. vivax* parasites resistant to chloroquine and the effectiveness of antifolates in vivax malaria treatment together with a correlation between mutations in *P. vivax dhfr* and *dhps* genes and SP treatment failure, the point mutations in these genes were investigated.

**METHODS:**

Blood samples from 54 patients experiencing vivax malaria symptomatic episodes in the Amazonian Region were investigated. Genomic DNA was extracted using a DNA extraction kit (QIAGEN^TM^). Nested polymerase chain reaction (PCR) amplification was carried out followed by Sanger sequencing to detect single nucleotide polymorphisms (SNPs).

**FINDINGS:**

All tested isolates showed non-synonymous mutations in *pvdhfr* gene: 117**N** (54/54, 100%) and 58**R** (25/54, 46%). Double mutant allele 58**R**/117**N** (F**R**T**N**I, 28%) was the most frequent followed by triple mutant alleles (58**R**/117**N**/173**L**, F**R**T**NL**, 11%; 58**R**/61**M**/117**N**, F**RMN**I, 5% 117**N**/173**L**, FST**NL**, 4%) and quadruple mutant allele (58**R**/61**M**/117**N**/173**L**, F**RMNL**, 2%). A single mutation was observed at codon C383**G** in *pvdhps* gene (S**G**KAV, 48%).

**CONCLUSION:**

No evidence of molecular signatures associated with *P. vivax* resistance to SP was observed in the Brazilian samples.

*Plasmodium vivax* is the most geographically widespread human malaria parasite. It is prevalent mainly outside Africa including Asia, South and Central America, and the Middle East. In the Americas, the burden of vivax malaria mostly affects Venezuela and Brazil. In Brazil, malaria transmission occurs almost entirely (> 99% of the registered cases) within the northern Brazilian Amazon Region where both *P. falciparum* and *P. vivax* infections co-exist. In this area, *P. vivax* is the predominant species, responsible for 89% of 194,409 malaria cases reported in 2017.^1^ Nowadays, falciparum malaria is treated with a 3-day fixed Artesunate+Mefloquine combination, according to Brazilian National Malaria Program guidelines, and a radical cure for *P. vivax* malaria is achieved with 25 mg/kg of CQ base for three days (maximum adult dose, 1.5 g for three days), combined with a short hypnozoitocidal regimen of 0.5 mg/kg/day of primaquine (PQ) base (maximum daily dose, 30 mg/day) for seven days in patients that weighed below 70 kg. As subtherapeutic PQ doses may lead to relapse in overweight patients, weight-adjusted PQ doses are now recommended in Brazil for patients over 70 kg.

*P. falciparum* resistance to chloroquine (CQ) observed in the 1980s greatly contributed to the emergence of falciparum malaria outbreaks across Amazon.^2^ *P. vivax* resistance to CQ occurred later in 1989 in Papua New Guinea^3^ and CQ monotherapy was ineffective. Following this seminal observation, numerous cases of CQ resistance were reported in Southeast Asia^4^ and South America,^5^ ^,^ ^6^ thus complicating the current international efforts for malaria control and elimination, and signalling the need for alternative drugs for vivax malaria treatment.

Antifolates, most notably sulfadoxine-pyrimethamine (SP), have been used as anti-malaria for *P. falciparum* treatment throughout the world because this combination is inexpensive, relatively safe, and requires only a single dose course treatment. SP had been available in Brazil since 1960s to treat CQ-resistant falciparum malaria but SP-resistant *P. falciparum* isolates appeared since 1990; SP is not used for malaria therapy in Brazil. Although resistant to antifolates, *P. falciparum* treatment has been well documented in many parts of the world, and *P. vivax* chemoresistance to SP is scarcely studied.

Sulfadoxine and pyrimethamine are competitive inhibitors of dihydropteroate synthase (*dhps*) and dihydrofolate reductase (*dhfr*), the two major proteins involved in folate biosynthesis pathway^7^. Polymorphisms in these two genes are the major factors associated with SP resistance.

Data on *pvdhfr* and *pvdhps* genotypes are available for many Southeast Asian countries. Such reports remain limited for some *P. vivax* endemic areas, notably South America. In Brazil, only one study characterising polymorphisms in *pvdhfr* gene was documented^8)^ and there is no report on the frequency of single nucleotide polymorphism (SNP) in *dhps* gene in *P. vivax* clinical isolates from Brazilian endemic areas.

Given the emergence of *P. vivax* CQ resistant parasites and the effectiveness of antifolates in malaria vivax treatment together with a strong correlation between mutations in *P. vivax dhfr* and *dhps* genes and SP treatment failure,^9^ the present paper reports an investigation on the pattern of point mutations in *pvdhfr* and *pvdhps* genes in Brazilian isolates.

## MATERIALS AND METHODS

*Parasites isolates and DNA extraction* - Blood samples from Amazon Region (Acre, Amapá, Amazonas, Rondônia and Pará) were collected from 54 patients presenting with vivax malaria from 2010 to 2016 at the Laboratório de Doenças Febris Agudas, INI-IPEC, Fiocruz, the Reference Clinical Laboratory for Malaria in the Extra-Amazon to the Brazilian Ministry of Health. All the clinical isolates were diagnosed as single *P. vivax* infections by light microscopic examination of Giemsa’s solution-stained blood smears and by *P. vivax* cysteine-proteinase target gene polymerase chain reaction (PCR).^10^ The parasitaemia ranged from 960 to 19160 parasites/µL. All malaria patients presented with clinical signs and/or symptoms of uncomplicated malaria, such as fever, headache, and chills, and the baseline characteristics were similar. No significant difference in parasitaemia was observed among the studied Brazilian localities and all the Brazilian endemic states were hypoendemic malaria areas.

Genomic DNA was extracted using a commercially available DNA extraction kit (QIAGEN^TM^, Frankfurt, Germany), following the manufacturer’s instructions. This study was performed according to the protocols previously approved by the Ethical Research Committees of Fiocruz (32839013.6.00005248). Patients were treated with CQ plus PQ, according to the Brazilian Ministry of Health recommendation for uncomplicated vivax malaria treatment and were followed up to 42 days. No treatment failure was detected during this period.

*Nested PCR and electrophoresis* - Nested PCR amplification of *pvdhfr* and *pvdhps* were carried out as described previously.^11^ Ten point mutations were investigated: F57**L**/**I**, S58**R**, T61**M**, S117**T**/**N** and I173**F**/**L** for *pvdhfr*, and S382**A**, C383**G**, K512**M**/**T**/**E**, A553**G** and V585**G** for *pvdhps*. PCR products were analysed by ethidium bromide-stained agarose-gel (2%) electrophoresis.

*DNA sequencing and SNPs detection* - The 632 bp and 767 bp fragments generated by amplification of *pvdhfr* and *pvdhps*, respectively, were extracted and purified from gel using the Wizard® SV Gel and PCR Clean-Up System (Promega, Madison, WI, USA) commercial kits. Briefly, the amplified fragments were sequenced using BigDye Terminator cycle sequencing ready reaction version 3.1 and ABI Prism DNA analyser 3730 (Applied Biosystems) at the Genomic Platform/PDTIS/Fiocruz.^12^ The direct DNA sequencing from PCR products were compared with the reference Sal I sequence of *pvdhfr* (GenBank X98123) and *pvdhps* (GenBank AY186730.1). Forward and reverse sequences were analysed using the free software, Bioedit Sequence Alignment Editor version 7.2.5. PCRs and DNA sequencing were randomly repeated to check possible sequence errors introduced during these stages.

## RESULTS

All the 54 isolates sequenced for *pvdhfr* gene showed non-synonymous mutations: 117**N** (54/54; 100%) and 58**R** (25/54; 46%) mutant alleles were more frequent, while 173**L** (9/54; 17%) and 61**M** (4/54; 7%) were detected at lower frequencies. Mutation at position 57**L** was not found (Table I). The most common single mutant allele was 117**N** (27/54; 50%). This single mutant was more frequent in Acre (10/15; 66%), Amazonas (11/23; 52%) and Pará states (4/8; 50%), compared to Rondônia state (1/7; 14%), where double 58**R**+117**N** mutant was dominant (Table II). Independent of the year collection, Amazonas state showed the highest number of *pvdhfr* gene mutations (23/54; 42,5%), followed by Acre (15/54; 27,7%, Para (8/54; 15%) and Rondônia (7/54, 13%) (Tables III-VI). Apparently in 2011, Acre presented more *pvdhfr* gene mutations (7/15; 47%) than Amazonas (2/23; 8,6%) (Tables III-IV), but this difference could be related to the smaller number of Amazonas samples collected in 2011, because when percentages are compared instead of figures, 100% of Amazonas (2/2) and Acre samples (7/7) presented mutations in 2011.

The double 58**R**/117**N** allele (F**R**T**N**I, 28%) was the most common allele, contrasting with the frequencies of other *dhfr* double, triple, or quadruple mutant alleles, with lower frequencies: 58**R**/117**N**/173**L** (F**R**T**NL,** 11%), 58**R**/61**M**/117**N** (F**RMN**I, 5%), 117**N**/173**L** (FST**NL**, 4%), and 58**R**/61**M**/117**N**/173**L** (F**RMNL**, 2%). In all localities, wild-type *pvdhfr* (FSTSI) was not observed (Table VII). The 58**R/**117**N** double mutant allele was detected in Acre (2/15; 13%), Rondônia (5/7; 71%), and Amazonas (8/23; 35%) while the 117**N**+173**L** only in Pará and Rondônia. The triple mutant allele 58**R/**117**N/1**73**L** was found in all localities, except Rondônia, and the quadruple mutant 58**R**/61**M**/117**N**/173**L** was observed only in one isolate collected from Amazonas state (1/23; 4%) (Table VIII). The frequencies of double, triple, or quadruple mutants were not related to the year of collection (Tables III-VI).

**TABLE I:** Plasmodium vivax dhfr and dhps amino acid changes in 54 P. vivax isolates from Brazilian endemic areas

Gene	SNPs	Prevalence N (%)
*dhfr*	58**R**	25 (46)
	61**M**	4 (7)
	117**N**	54 (100)
	173**L**	9 (17)
*dhps*	383**G**	26 (48)

SNPs: single nucleotide polymorphisms.

**TABLE II:** Number of alleles in dhfr and dhps genes observed among 54 Brazilian Plasmodium vivax isolates, according to sampling location

Gene	SNPs	Amazonas (n = 23)	Acre (n = 15)	Amapá (n = 1)	Pará (n = 8)	Rondônia (n = 7)
*dhfr*	117**N**	12	10	-	4	1
	58**R/**117**N**	8	2	-	-	5
	117**N/** 173**L**	-	-	-	1	1
	58**R**/117**N**/173**L**	1	3	1	1	-
	58**R**/61**M/**117**N**	1	-	-	2	-
	58**R**/61**M/**117**N/**173**L**	1	-	-	-	-
*dhps*	383**G**	13	8	-	5	-

SNPs: single nucleotide polymorphisms.

**TABLE III:** Number of alleles in dhfr and dhps genes observed among 23 Plasmodium vivax isolates from Amazônia, according to year of blood collection

Genotype	Mutation codon	2010	2011	2012	2013	2015	2016	Total
*Dhfr*	117**N**	5	-	1	-	4	2	12
	58**R**/117**N**	1	2	1	-	3	1	8
	58**R**/117**N**/173**L**	-	-	-	1	-	-	1
	58**R**/61**M**/117**N**	-	-	1	-	-	-	1
	58**R**/61**M**/117**N**/173**L**	-	-	1	-	-	-	1
	TOTAL	6	2	4	1	7	3	23
*Dhps*	383**G**	5	-	4	1	2	1	13

Concerning *pvdhps* gene in 26 out of 54 (48%) isolates only a single mutation at codon C383**G** was detected. No other mutations, including 382**A**, 512**M**, 553**G**, and 585**C**, were found. The wild-type SCKAV (52%) and single haplotype S**G**KAV (48%) were observed at similar frequencies. The single mutant 383**G** was observed in isolates from Amazonas (13/23, 56%), Acre (8/15, 53%) and Pará (5/8, 62%) but not in isolates from Rondônia state (0/7) (Table II). Once again frequencies of *pvdhps* gene mutations were not related to the year of collection (Tables III-VI).

Combining *pvdhfr* and *pvdhps* alleles, only one haplotype (F**R**T**N**I for *pvdhfr* and S**G**KAV for *pvdhps*) was seen in three of the four study sites with a higher frequency in Amazonas state (where one *pvdhfr* quadruple mutant was detected) (Table IX). No *pvdhfr* or *pvdhps* quadruple or quintuple mutant haplotype, which might result in poor clinical response against antifolate drugs, was detected in any of the Brazilian localities investigated.

## DISCUSSION

Mutations in *pvdhfr* and *pvdhps* genes have been found to be associated with antifolate drug resistance. Both *in vivo* ^13^ and *in vitro* assays suggested that these molecular markers may provide information about the trends of SP resistance in *P. vivax*. Here, we investigated SP resistance in vivax isolates by seeking specific point mutations in *pvdhfr* and *pvdhps* genes.

It has been postulated that *pvdhfr* 117**N** mutation might occur first, followed by S58**R** mutation.^14^ In this study, *pvdhfr* S117**N** was detected in all isolates followed by 58**R** (74%), 173**L** (17%), and 61**M** (7%) polymorphisms, supporting that S117**N** mutation is the first step in drug selection process. These data are similar to other observations done in areas where *P. falciparum* and *P. vivax* parasites co-exist.^14^ ^,^ ^15^

The predominance of S117**N** followed by the double mutant 58**R/**117**N** (28%) was also analogous to those reported in India,^15^ Afghanistan,^16^ China,^17^ Nepal,^18^ Thailand,^19^ Colombia,^20^ ^,^ ^21^ French Guiana^19^ and Brazil.^8^ The triple 58**R/**117**N/**173**L** *pvdhfr* mutant, not seen in *P. vivax* samples from Southeast Asian, where non-synonymous mutation in codon 173 comprises the change of I by F generating the 173**F** allele, was here detected in Amazonas, Acre, Amapá and Pará states and also in *P. vivax* parasites from French Guiana^19^ ^,^ ^22^ and Amazonas, Brazil.^8^ Conversely, the non-synonymous mutation at position F57**L** not recorded in this study was exclusively reported in Southeast Asian samples; findings that could reflect different drug pressure history and selective processes in the old and new worlds. In fact, the genetic similarity of 173**L** SNP recorded for *P. vivax* parasites from two neighbouring South-American countries Brazil and French Guiana,^19^ reinforce the possible existence of geographic subdivision of different*P. vivax* parasites in samples from the old and new worlds.

**TABLE IV:** Number of alleles in dhfr and dhps genes observed among 15 Plasmodium vivax isolates from Acre, according to year of blood collection

Genotype	Mutation codon	2011	2013	2014	2015	2016	Total
*Dhfr*	117**N**	4	-	-	4	2	10
	58**R**/117**N**	1	-	1	-	-	2
	58**R**/117**N**/173**L**	2	1	-	-	-	3
	TOTAL	7	1	1	4	2	15
*Dhps*	383**G**	5	-	-	2	1	8

**TABLE V:** Number of alleles in dhfr and dhps genes observed among eight Plasmodium vivax isolates from Pará, according to year of blood collection

Genotype	Mutation codon	2010	2011	2013	2015	2016	Total
*Dhfr*	117**N**	1	-	-	1	2	4
	117**N**/173**L**	-	1	-	-	-	1
	58**R**/117**N**/173**L**	-	-	1	-	-	1
	58**R**/61**M**/117**N**	-	-	-	2	-	2
	TOTAL	1	1	1	3	2	8
*Dhps*	383**G**	1	-	-	1	2	5

Concerning the *pvdhps* gene, previous data indicated that mutations were mainly detected at codons A383**G** and A553**G** ^14^ ^,^ ^21^ ^,^ ^23^ ^,^ ^24^ and suggested that these mutations alone could be responsible for reduced sensitivity to sulfa and sulfones.^25^ ^,^ ^26^ In the present work, the wild-type (52%) and the mutated codon 383**G** (48%) were detected at similar frequencies among *P. vivax* isolates, similar to reports from Thai-Cambodian (53%),^7^ Thai-Myanmar border (47%)^27^ and Indonesia (50%).^9^ Whereas, in a Colombian study investigating polymorphisms in *pvdhps*, the wild-type was the most frequently detected (71.6%);^21^ the same was true in India (79%)^15^ and also in Thai - Cambodian border (74%). Therefore, the *pvdhps* wild-type allele seems to be common in malaria endemic areas of the world, probably due to a low SP drug selection in the sympatric *P. vivax* populations of these countries. However, in Brazil, for example, SP or its analogues have been used for fever and antimicrobial therapy and, in this way, there continues to be a lengthy selection pressure for SP-resistant strains of *P. vivax* resulting to low frequencies of wild-type *pvdhps* parasites.

Amazonas state recorded the highest number of *pvdhfr* and *pvdhps* mutations. This finding could not be attributed to differences of antimalarial drug usage in Brazilian states because the malaria treatment in Brazil is the same all over the country. Besides that, SP has never been recommended for vivax malaria treatment and SP has been excluded from *P. falciparum* treatment since 1989. Thus, it is more reasonable suppose that more mutations were found in Amazonas due to the highest number of samples examined from this locality, as only one sample from Amazonas was from a border area of the Amazon with Acre - the second state that showed the greatest number of mutations. A study with a representative number of Amazonian state cases may help answer this question.

**TABLE VI:** Number of alleles in dhfr gene observed among seven Plasmodium vivax isolates from Rondônia, according to year of blood collection

Genotype	Mutation codon	2010	2011	2014	Total
*Dhfr*	117**N**	1	-	-	1
	58**R**/117**N**	-	2	3	5
	117**N**/173**L**	-	-	1	1
	TOTAL	1	2	4	7

**TABLE VII:** Deduced dhfr and dhps haplotype profiles in 54 Plasmodium vivax isolates from Brazilian endemic areas

Gene	Haplotypes	N	%
*dhfr*	FST**N**I	27	50
	F**R**T**N**I	15	28
	FST**NL**	2	4
	F**R**T**NL**	6	11
	F**RMN**I	3	5
	F**RMNL**	1	2
*dhps*	S**G**KAV	26	48
	SCKAA (wild type)	28	52

Bold letters: mutated codons; F: codon 57; S: codon 58; T: codon 61; S: codon 117; I: codon 173.

**TABLE VIII:** Prevalence of Plasmodium vivax dhfr and dhps amino acid changes in 54 P. vivax isolates from Brazilian endemic areas

Gene	Mutants	SNPs	Prevalence N (%)
*dhfr*	Single	S117**N**	27 (50)
	Double	S58**R/**S117**N**	15 (28)
		S117**N/** I173**L**	2 (4)
	Triple	S58**R**/S117**N**/I173**L**	6 (11)
		S58**R**/T61**M/**S117**N**	3 (5)
	Quadruple	S58**R**/T61**M/**S117**N/** I173**L**	1(2)
*dhps*	Single	C383**G**	26 (48)

SNP: single nucleotide polymorphisms.

**TABLE IX:** Percentage of double mutant dhfr / single mutant dhps in 54 Plasmodium vivax samples according to Brazilian states

Amazônia	Acre	Pará
NP (%)	NP (%)	NP (%)
13 (24)	8 (15)	5 (9)

NP: number of positive.

In conclusion, we found no molecular strong evidence of *P. vivax* SP resistance in recently collected Brazilian samples. As mutations in *P. vivax dhps* and *dhfr* genes provide a valuable tool for epidemiological surveillance of SP resistance, the prevalence of point mutations on these genetic markers of SP resistance should be assessed for providing information for future treatment policy with alternative antifolate drugs because of the appearance and dispersion of CQ resistance in malaria endemic areas.
